# BeerDeCoded: the open beer metagenome project

**DOI:** 10.12688/f1000research.12564.2

**Published:** 2017-10-20

**Authors:** Jonathan Sobel, Luc Henry, Nicolas Rotman, Gianpaolo Rando

**Affiliations:** 1 Hackuarium Association, Renens, Switzerland

**Keywords:** metagenomic, beer, citizen science, crowdfunding

## Abstract

Next generation sequencing has radically changed research in the life sciences, in both academic and corporate laboratories. The potential impact is tremendous, yet a majority of citizens have little or no understanding of the technological and ethical aspects of this widespread adoption. We designed BeerDeCoded as a pretext to discuss the societal issues related to genomic and metagenomic data with fellow citizens, while advancing scientific knowledge of the most popular beverage of all. In the spirit of citizen science, sample collection and DNA extraction were carried out with the participation of non-scientists in the community laboratory of Hackuarium, a not-for-profit organisation that supports unconventional research and promotes the public understanding of science. The dataset presented herein contains the targeted metagenomic profile of 39 bottled beers from 5 countries, based on internal transcribed spacer (ITS) sequencing of fungal species. A preliminary analysis reveals the presence of a large diversity of wild yeast species in commercial brews. With this project, we demonstrate that coupling simple laboratory procedures that can be carried out in a non-professional environment with state-of-the-art sequencing technologies and targeted metagenomic analyses, can lead to the detection and identification of the microbial content in bottled beer.

## Introduction

Beer is probably the world’s oldest and most widely consumed alcoholic beverage on the planet, with a worldwide production of nearly 2 billion hectolitres (2·10E11 litres) annually [ The Barth Report, Hops 2015/2016], and, as DNA sequencing becomes increasingly cheap, whole genome sequencing and metagenomic analyses are being explored as tools to better understand brewing in particular, and food fermentation in general ^1^. Complex microbial communities influence the wine- and cheesemaking process throughout ^2, 3^. Indeed, microbial communities contribute to nutritional and aromatic properties, as well as shelf life of the products. In the case of wine, microorganisms are present in the soil, on the grapes, and in the fermenter, being carried over from the vine to the must to the wine, and there is increasing evidence for the existence of an important microbial contribution to the notion of “terroir” (i.e regional environmental factors that affect the properties of the final product) ^4– 7^. One question that remains unanswered is whether there is such a thing as a “terroir” for beer.

Of particular interest is sour beers, such as lambic and gueuze, beverages produced without the controlled addition of known yeast cultivates. Instead, the wort is exposed to ambient air, allowing naturally occurring bacteria and yeasts to start the fermentation and leading to a production that is difficult to standardize. To our knowledge, three initiatives are currently exploring the role of the beer microbiome in the brewing process and how it shapes the characteristics of the final product. Using metagenomic analyses, Kevin Verstrepen and colleagues at KU Leuven, Belgium, study the production of lambic, a traditional Belgian beer produced by spontaneous fermentation [ VIB project 35]. Similarly, Matthew Bochman and colleagues at Indiana University, USA, have recently published preliminary results showing how the microbial community evolved over the fermentation process, together with the relative abundance of the organic acids that give sour beer its characteristic taste ^8, 9^. Similarly, researchers at the University of Washington, USA, have studied open-fermentation beer and discovered a novel interspecific hybrid yeast ^10^.

To investigate the microbial composition of a collection of commercial beers, we initiated BeerDeCoded in the context of Hackuarium, a Swiss not-for-profit organisation that supports unconventional research projects and promotes the public understanding of science. Members of the Hackuarium community are interested in participatory biology and want to promote interdisciplinary citizen research and innovation outside traditional institutions, using low-cost, simple and accessible technologies. The goal of the BeerDeCoded project is not only to broaden the scientific knowledge about beer, but also to improve the public understanding of issues related to personal genomics, food technology, and their role in society. With the release of this first data set, we built the proof of concept for a targeted metagenome analysis pipeline for beer samples that can be used in high schools, citizen science laboratories, craft breweries or industrial plants.

## Methods

### Beer sample preparation

The content of each beer sample was mixed to homogeneity by inversing the bottle several times. 50 mL were transferred into a conical tube and centrifuged (5000 rpm, 20 min, 4°C) to collect cells and other precipitable material. Pellets were resuspended with 1 mL TE buffer (Tris 10 mM, EDTA 1 mM, pH 8.0) and transferred into 1.5 mL tubes. The samples were centrifuged (10000 rpm, 10 min, 4°C), the supernatant was removed and the pellet stored frozen (-20°C) until future analyses. The ZR Fecal DNA MiniPrep kit (Zymo Research) was used for DNA extraction with minor modifications to the original protocol ^11^. Sludge pellets were used instead of the 50-100 mg of fecal material suggested by the manufacturer..

### Quality control for DNA extraction

To ensure the DNA was free from proteins and other contaminants, the absorbance of DNA samples was measured at 230, 260 and 280 nm using a NanoDrop 2000 spectrophotometer (Thermo Fisher Scientific).

### ITS amplification

Yeast genomic DNA was amplified using the fungal hypervariable region ITS1 (internal transcribed spacer 1) as previously described ^11^ using the following primers: BITS (5’–CTACCTGCGGARGGATCA–3’) and B58S3 (5’– GAGATCCRTTGYTRAAAGTT–3’). Typical PCR reactions contained 5–100ng of DNA template. Amplicon size (500nt) was verified using gel electrophoresis and with a fragment analyser. ITS amplicons were purified using AM-Pure XP beads following the manufacturer’s instructions (Beckman Coulter). Dual indices and Illumina sequencing adapters were attached using the Nextera XT Index Kit following manufacturer’s instructions (Illumina).

### Sequencing

MiSeq sequencing was performed using the MiSeq v3 reagent kit protocol (Illumina). Briefly, the amplified DNA was quantified using a fluorimetric method based on ds-DNA binding dyes (Qubit). Each DNA sample was diluted to 4 nM using 10 mM Tris pH 8.5 and 5 uL of diluted DNA from each library were pooled. In preparation for cluster generation and sequencing, 5 uL of the pooled final library was denatured with 5 uL of freshly diluted 0.2 N NaOH and combined with 30% PhiX control library to serve as an internal control for low-diversity libraries. After loading the samples on the MiSeq, paired 2x 300bp reads were generated and exported as FASTq files.

### Bioinformatics analysis

The curated set of ITS sequences from the Refseq database ( Targeted Loci) was used to build an ITS index for the Burrows-Wheeler Aligner (BWA, version 0.7.13) ^12^. The BWA was used with standards parameters to map the paired-end reads of each beer from the fastq files to our ITS index. The BAM files were sorted and indexed using samtools ^13^. A quality control of the BAM files was performed using SAMstat (version 1.5) ^14^. A read quality threshold above 3 (MAPQ score) was applied in order to remove low quality and non-unique mapping reads. Subsequently, the number of ITS per beer and per species were counted and only species with over 10 reads were taken into consideration. Visualization of the results were performed with R (version 3.4.0).

## Results

Over the month of June 2015, a total of 124 individuals contributed over 10,000 Euros to a crowdfunding campaign that provided financial resources for the first stage of the BeerDeCoded project. Reaching out to the public through this campaign also enabled crowdsourcing a collection of 120 beer samples from 20 countries. We have subsequently demonstrated that it is possible to extract DNA directly from bottled beer using low cost methodologies, typically available to citizen scientists (see Methods).

The internal transcribed spacer regions (ITS) of fungal species ^15^ were then amplified and, after quality control, 39 samples were sent for DNA sequencing. These 39 commercial beers originated from 5 different countries: 30 were from Switzerland, five from Belgium, two from Italy, one from France and one from Austria. We obtained an average library size of 600K reads (min 350K, max 2400K see Table 1) with more than 99% of reads mapping to the ITS database per sample.

**Table 1.** Sequencing libraries statistics.

Beer library	total read count	unmapped read count	mapping percentage [%]
Ambree des Brigands du Jorat	645239	20674	99,97
Bieraria Tschlin BE	640291	15700	99,98
Brasserie dAyent Celsius Folamour	634121	17162	99,97
Brasserie des 5 quatre mille Biere de Zinal	600066	16194	99,97
Brasserie du Griffon La Fourbe	377774	9592	99,97
Brasserie du Vieux Chemin La Prudencia	454462	12490	99,97
Brasserie DuPont BioLegere	483889	12486	99,97
Brasserie Gessienne Blanche	379492	6164	99,98
Brasserie Sierrvoise Noire	353605	14524	99,96
Brasserie Tardiv	585357	18061	99,97
Brasseurs de Volleges La Tourbillon	418709	11948	99,97
Calvinus Blanche	473262	10944	99,98
Chimay Red Cap	552594	7806	99,99
Chimay Tripel	587167	11089	99,98
Coudres Blonde	652259	36080	99,94
Coudres Pale Ale	431653	16170	99,96
Delirium Tremens	627271	10432	99,98
Docteur Gabs Houleuse	681220	25303	99,96
Docteur Gabs Pepite	597756	10987	99,98
Docteur Gabs Tempete	644890	8640	99,99
Hackuarium Fakufaku	489232	9443	99,98
Homebrew Amber Ale	578714	12711	99,98
Homebrew Roter Baron	350211	17288	99,95
Homebrew SquareBeer	421463	8670	99,98
Hoppy Couple	612653	14486	99,98
La Cotta Bionda	681507	16781	99,98
La Montheysanne	381861	11297	99,97
La Mule Browney	402023	6068	99,98
La Nebuleuse ChichaBeer experimental	670287	18244	99,97
La Nebuleuse Embuscade	591798	22089	99,96
La Nebuleuse Malt Capone	637362	15770	99,98
La Nebuleuse Stirling	681512	29075	99,96
La Salamandre	643011	18059	99,97
Les Muraille Pieuse	391583	11006	99,97
Mateo 21	392582	7935	99,98
Orval	524342	21694	99,96
Trois Dames	2600874	78441	99,97
Valaisanne Amrich	368145	8278	99,98
Waldbier 2014 Schwarzkiefer	350005	16224	99,95

**Figure 1.**
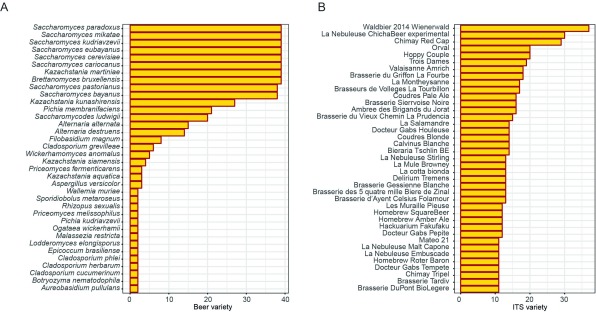
Barplot graph representing. ( **A**) the number of beers containing the species (n=36) occurring in at least two samples. Species (n=52) present in only one sample were excluded for clarity. ( **B**) represents the number of fungal species identified in each of the 39 bottled beers.

**Figure 2.**
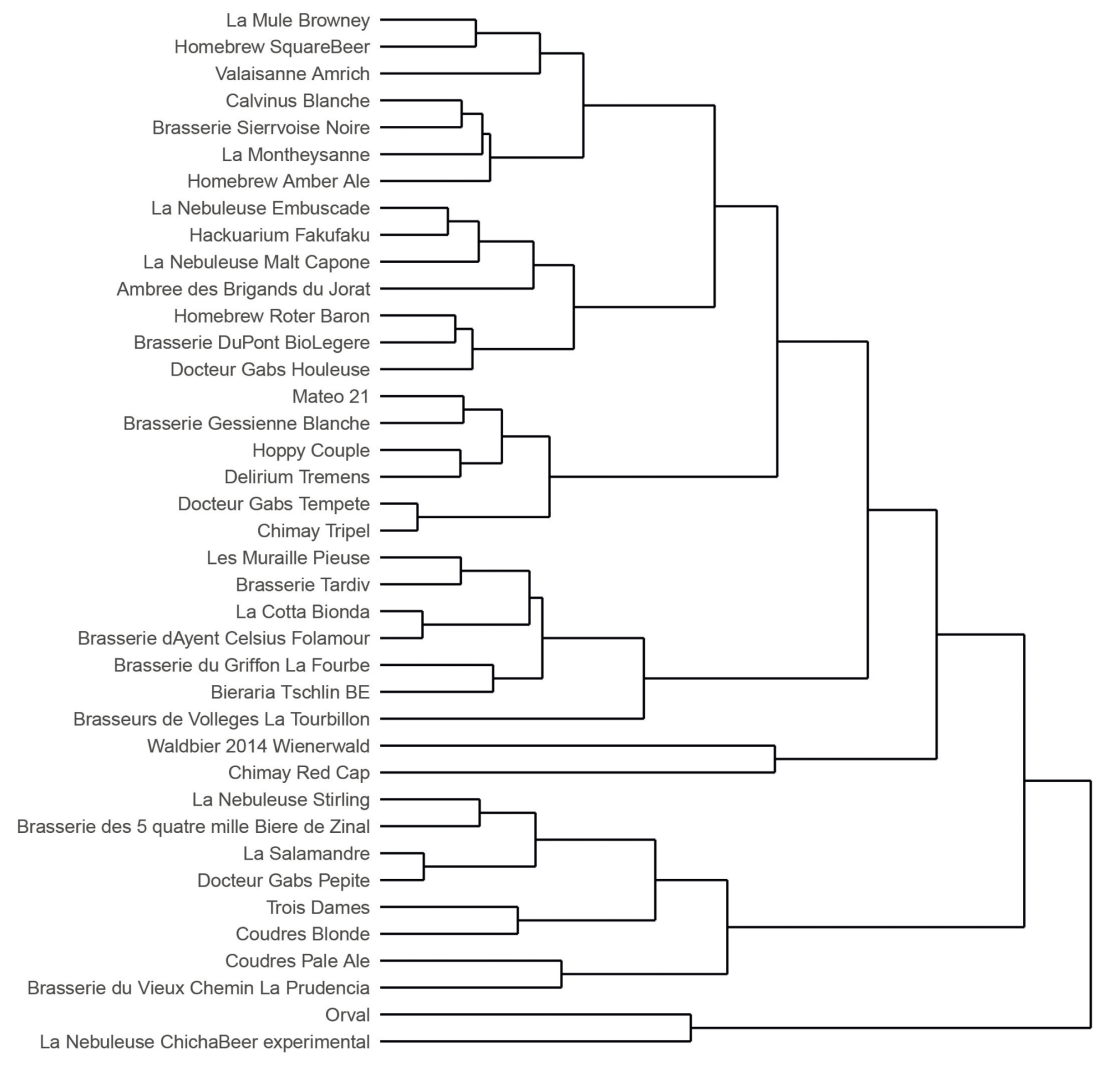
Hierarchical clustering of the 39 beers included in this study, based on their fungal content. We applied the Ward’s method on the Euclidean distance computed on the log10 counts matrix.

A total of 42 fungal species were identified, 24 of which were present only in a single brew. This high variety of wild yeasts in commercial beers was unexpected ( Figure 1 A), with some brews containing traces of up to more than 10 different fungal species ( Figure 1 B). The beer in which we measured the highest ITS diversity (19 fungal species) was Waldbier 2014 Schwarzkiefer, an Austrian beer brewed using pine cones collected in local forests. Two other beers contained more than 12 fungal species: La Nébuleuse Cumbres Rijkrallpa (a sour/wild ale beer made with cranberries and the fermented corn “Chicha”) and Chimay Red Cap, a Belgian trappist beer. Using hierarchical clustering, we built a proximity tree of the different beers ( Figure 2).

**Figure 3.**
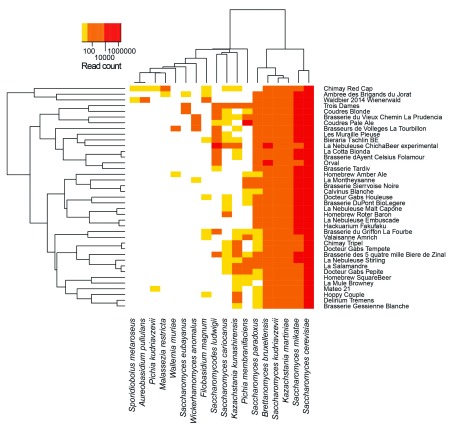
Heatmap of the number of reads per ITS per beer. Only ITS with more than 10 reads and present in at least two beers are shown.

Consistent with its widespread use for fermentation, brewer’s yeast ( *Saccharomyces cerevisiae*) was detected in all beer samples, accounting for between 11% (Orval, an ale beer by Belgian Brasserie d’Orval) and 99% (Tempête, an ale from the Swiss brewery Docteur Gab’s) of all sequencing reads. In most samples, *S. cerevisiae* was present at very high levels (typically 90–97% of reads, Figure 3). More surprisingly, *Saccharomyces mikatae*, a species used in winemaking ^16^ was also relatively abundant in all samples (0.5–5%). Interestingly, most brews were found to contain low to medium abundance of multiple other yeast species, including *Saccharomyces kudriavzevii* and *Saccharomyces eubayanus* (a probable parent of *Saccharomyces pastorianus*) and *Brettanomyces bruxellensis* (typically used for the production of the Belgian beers). Non-conventional, as well as wild yeast, such as *Saccharomyces cariocanus* and *Saccharomyces paradoxus*, two species closely related to *Saccharomyces cerevisiae* were also found. Another example is *Kazachstania sp.*, a wild yeast of commonly found in brines ^17^. The presence of this species may be of interest, as it was previously reported that adding the parent *Kazachstania servazzi* to the brewing process 24 hours before the ale yeast contributed to the production of high level of esters, producing a strong fruity and floral aroma ^18^.

## Discussion and future perspectives

While a continuous process of market consolidation has lead to 5 companies controlling more than half of global beer production, there has been an explosion of craft industries over the past years, especially in Europe and North America. In 1978 there were 89 large industrial breweries in the USA. In 2016, there were 5,301, among them 3,132 small, independent microbreweries ( American Brewers Association). There is a parallel with Hackuarium, an independent “craft” science initiative that has branched out from large institutional research institutes and provides an environment that allows scientists to explore topics that are rarely found in academia or industry. What is truly unique is the participation of individuals with no formal science training, and therefore the strong focus on citizen science and communication. With the BeerDeCoded project, we explored the potential of crowdfunding and crowdsourcing in engaging members of the general public in the production of scientific knowledge. We demonstrated that it is possible to execute complex molecular analyses on everyday products using limited resources and technical support from research institutions, and no financial support from traditional funding sources. The resulting dataset contains the ITS profile of 39 bottled beers from five different countries, revealing the low abundance but widespread presence of wild fungal species. It is a proof of concept that sequencing beer metagenomic information can be done, at least partly, with the help of the public. For the current analysis, we relied on high-throughput sequencing technology available to us through a partnership, a technology that may be out of reach for individuals working in non-traditional research environments. In the future, we would like to overcome this limitation, for example by providing a pipeline based on portable sequencing technologies, such as Oxford Nanopore’s minION instrument. Further analyses could also go as far as shedding light on the so-called biological ”dark matter” of the beer ecosystem ^19, 20^.

With the costs of DNA sequencing falling dramatically, and with the emergence of portable and user-friendly instrumentation, we believe that it is a favorable time to expand the application of DNA analysis to novel fields, including food and beverage. This industry is starting to explore the potential of genome sequencing to understand the contribution of various species to product characteristics. The sequencing of the full genome of 157 brewing yeast strains was, for example, recently reported ^21^. Metagenomic analyses could also have important implications for the optimization and batch-to-batch reproducibility of the various fermentation processes, as well as quality control, traceability and authentication of the products. One hypothesis that could be investigated further in the future is whether the presence of a specific fungal species can be diagnostic for a unique geographic area. In our data set, the non *Saccharomyces* yeast that contributes to wine aroma through the production of volatile compounds, *Wickerhamomyces anomalus*, was found exclusively in five of the brews manufactured in Switzerland. The limited sample size, however, does not allow us to draw a statistically significant conclusion, and it remains to be seen if *W. anomalus* is present in beers from other locations as well. Due to inherent limitations of DNA sequencing, it is difficult to anticipate whether the microbes identified are likely to be having an impact on the fermentation process. However, based on the identification of strains present in brews with desired characteristics, controlled experiments in which the microbial composition of the brew is altered could allow us to investigate if the presence of specific microorganisms affects flavour ^22^. The origin of each yeast species could also be investigated; i.e. whether they come with the ingredients or from the environment at the production site. Techniques to sample airborne DNA exist ^23^. Furthermore, other protocols could also be used to catalogue plant DNA ^24^, such as malt and hop varieties, and to map the bacterial diversity.

In order to standardize and simplify our pipeline, and facilitate the contribution of new data and their further analysis by individuals not involved in this initial study, we are in the process of developing a BeerDeCoded repository and a Galaxy instance ^25^. This tool will enable any citizen scientist to carry out beer metagenomics and reproduce our analysis. In the meantime, we encourage researchers from other laboratories, microbreweries and citizen laboratories to further explore our data set, and invite them to consider contributing additional data in the future.

## Data availability

The data referenced by this article are under copyright with the following copyright statement: Copyright: © 2017 Sobel J et al.

Data associated with the article are available under the terms of the Creative Commons Zero "No rights reserved" data waiver (CC0 1.0 Public domain dedication). 

The dataset contains the metagenomic profiles for 39 beers. The data was obtained using a targeted approach based on the phylogenetic typing with internal transcribed spacers (ITS) of ribosomal sequences. All methods, quality control, processed tables, metadata and code are accessible at: https://github.com/beerdecoded/Beer_ITS_analysis. The raw data are stored in the SRA database in the bio project PRJNA388541
