# Supplementary material for: BeerDeCoded: the open beer metagenome project
Source: F1000Res. 2017 Oct 20;6:1676. Originally published 2017 Sep 11. [Version 2] doi: 10.12688/f1000research.12564.2 (PMC5657021; doi:10.12688/f1000research.12564.2)
Supplement: Peer review history

# Peer review: Referee response for version 2

**DOI:** 10.5256/f1000research.13990.r27165

**Published:** 2017-11-02

**Authors:** Bastian Greshake

**Affiliations:** 1 Department for Applied Bioinformatics, Institute for Cell Biology and Neuroscience, Goethe University, Frankfurt am Main, Germany

The authors have satisfactory addressed the issues mentioned in the review of the first article version.

I have read this submission. I believe that I have an appropriate level of expertise to confirm that it is of an acceptable scientific standard.

# Peer review: Referee response for version 2

**DOI:** 10.5256/f1000research.13990.r27163

**Published:** 2017-11-02

**Authors:** Kristoffer Krogerus

**Affiliations:** 1 VTT Technical Research Centre of Finland Ltd., Espoo, Finland

I thank the authors for addressing all the concerns I brought up. However, while the post-alignment QC has reduced the amount of false positives, I'm still concerned about the lack of pre-alignment read trimming (which should be standard practice). The methods section or material on the project's github page make no mention of any trimming of the reads prior to alignment. As demonstrated in my first referee report, by trimming potential adaptor and primer sequences and removing poor quality bases at the ends of reads, the fungal diversity of the beers is reduced further (i.e. the amount of false positives are decreased), even with lenient post-alignment filtering (MAPQ < 3). I would recommend that the authors either redo the analysis once more with proper pre-alignment filtering as well, or clearly state in the results and discussion section that no such step was carried out and that the results may therefore contain a large number of false positives. For example, it is highly unlikely that all beers would contain traces of *Saccharomyces mikatae*, a yeast that to my knowledge never before has been isolated from fermentation environments and only ever from forest samples in Asia (1, 2). As demonstrated by the results in my previous referee report, *S. mikatae* is no longer detected in the samples I analyzed when pre-alignment quality trimming was performed.

I have read this submission. I believe that I have an appropriate level of expertise to confirm that it is of an acceptable scientific standard, however I have significant reservations, as outlined above.

## References

[1] NaumovGIJamesSANaumovaESLouisEJRobertsIN: Three new species in the Saccharomyces sensu stricto complex: Saccharomyces cariocanus, Saccharomyces kudriavzevii and Saccharomyces mikatae. *Int J Syst Evol Microbiol*.2000;50 Pt 5: 10.1099/00207713-50-5-1931 1931-42 10.1099/00207713-50-5-1931 11034507

[2] BellonJRSchmidFCaponeDLDunnBLChambersPJ: Introducing a new breed of wine yeast: interspecific hybridisation between a commercial Saccharomyces cerevisiae wine yeast and Saccharomyces mikatae. *PLoS One*.2013;8(4) : 10.1371/journal.pone.0062053 e62053 10.1371/journal.pone.0062053 23614011PMC3629166

# Peer review: Referee response for version 2

**DOI:** 10.5256/f1000research.13990.r27164

**Published:** 2017-10-20

**Authors:** Matthew L. Bochman

**Affiliations:** 1 Molecular and Cellular Biochemistry Department, Indiana University Bloomington, Bloomington, IN, USA

The authors have adequately addressed all of my concerns.

I have read this submission. I believe that I have an appropriate level of expertise to confirm that it is of an acceptable scientific standard.

# Peer review: Referee response for version 1

**DOI:** 10.5256/f1000research.13607.r26415

**Published:** 2017-09-28

**Authors:** Kristoffer Krogerus

**Affiliations:** 1 VTT Technical Research Centre of Finland Ltd., Espoo, Finland

This data note describes the fungal microbiome of 39 (commercial and homebrewed) beers as determined by next generation sequencing of ITS amplicons. The project was crowdfunded and many of the individual funders were also involved in providing beer samples and assistance during DNA extraction. While the results will be of interest, particularly for the brewing industry, I have some concerns with the analysis methods and the results presented in this first version of the manuscript.

**Major comments:**

 To determine the microbiome of the beers, the authors align the raw sequencing reads to a concatenated dataset containing fungal ITS sequences. To my understanding, no quality control or filtering was performed prior to and after the alignment. This will cause a large number of false positives, as many of the intragenic ITS sequences are very similar. To demonstrate, I repeated the analysis on six samples I retrieved from the NCBI SRA:

 SRR5740352: Chimay Red Cap

 SRR5740353: Chimay Tripel

 SRR5740362: Waldbier 2014 Wienerwald

 SRR5740364: La Nebuleuse Chicha

 SRR5740374: Orval

 SRR5740375: Trois Dames

 According to the results presented in the manuscript, each of the samples contained traces of at least 11 different species (see Figure 1 and Figure 3).

 What I did to the sequencing reads was

 1. Trim them using 'cutadapt' as follows (any similar tool would do the same job)

- Remove 20 first bases of each read
- Remove bases from end of read when quality score is less than 15
- Remove any reads shorter than 200 base pairs
- Approximately 80% of the bases were retained from each set of reads after these steps

2. After this the reads were aligned to the concatenated ITS sequence dataset using bwa mem with default settings as the authors had done. Reads mapping to the different sequences were then counted with the script used by the authors (obtainable from github). The results with no post alignment filtering: https://www.dropbox.com/s/llg94fgk23ag264/Beer_results_nofilter.txt

 3. After this I removed all reads that did not map to a unique location (i.e. could be mapped to the ITS sequences of several species) and reads where the two paired reads mapped to different sequences. This was done by removing all alignments with a MAPQ score below 4 and 'awk': https://www.dropbox.com/s/0iimh5fbb40qeh0/Beer_results_mapq4.txt

 As can be seen, the diversity is reduced considerably, and if all hits where the read count is less than 10 are also removed (as the authors had done), most samples now contain only *S. cerevisiae* and/or *B. bruxellensis*.

 4. The amount of false positives can be further reduced by filtering by a higher MAPQ score (e.g. 30): https://www.dropbox.com/s/3x4gyylsiykyu7o/Beer_results_mapq30.txt

 I therefore suggest that the authors redo the analysis with proper filtering to remove poor quality alignments and false positives in the results. The results and conclusions will subsequently have to be rewritten accordingly.

**Minor comments:**

 In the Methods section, under Beer sample preparation: I assume the DNA was extracted from the frozen yeast pellet? Any reason why it was not attempted to extract DNA from the beer itself, e.g. using the method described in reference (23)? This would allow analysis of filtered beers as well.

 Is Figure 2 necessary, since Figure 3 shows the clustering as well? Also, why does the clustering in Figures 2 and 3 differ? Were these generated using different clustering methodologies?

 It is mentioned that some of the beers contained speciality ingredients, such as pine cones. Do the authors know at what point in the production process these were added (i.e. pre- or post-boil)? This would have a large impact on how these ingredients affect the beer microbiome.

I have read this submission. I believe that I have an appropriate level of expertise to confirm that it is of an acceptable scientific standard, however I have significant reservations, as outlined above.

# Response

**Published:** 2017-10-10

**Authors:** Luc Henry

**Affiliations:** Hackuarium, Switzerland

Regarding the major comments:

 - We thank the referee for his thorough analysis of our results and for his comments. In our analysis, we initially choose to favor sensitivity over specificity and we did not filtered the reads based on their quality. We considered that, with 300 bp paired-end reads on ITS amplicons, we had enough specificity. But as referee 3 pointed out, we should take care of multiple mapping reads to for instance discriminate between the Saccharomyces sp. that are quite close to each other. Using a filter on MAPQ score > 30 is quite stringent, but the referee’s point on non-unique reads is critical. We have now re-performed the analysis using a series of MAPQ threshold. As expected, the higher the threshold, the more we lose some of the ITS detected, and some sensitivity. In our update of the article, we report results after using a filter with a MAPQ score of 3. This led to a reduction of the total fungal species identified, from 88 to 42, as well as of the unique occurrences, from 52 to 24.

 In addition we have performed quality control (QC) with samstat of our BAM files in order to see the quality score distribution of the reads of the alignment to the ITS database. The result of samstat has been added to our github repository. Samstat results show that about 3.2 % of reads are not aligned when reads with MAPQ score > 3 are taken into account. In addition we have checked the distribution of fragments size in the BAM files. We have an average fragment size of 383 bp with a sd of 10 bp. We have a very low fraction of fragments below 200 bp, or discordant pairs. Thanks to the comments of the Referee 3, we improved the specificity of the analysis and we may have excluded artifacts. Due to this re-analysis, we have updated Figure 1, Figure 2 and Figure 3, the code in our github repository as well as the main text. We have also added the QC informations.

 Regarding the minor comments:

 - With the goal to maximise our chances to obtain some preliminary results with the budget of the kickstarter campaign, we decided to use the pelleted material with the educated assumption that pelleted cells may protect DNA better than DNA left in solution. Reference (23) intrigued us: their DNA preparation method requires additional lyophilization, pulverization and digestion with amylase and we decided to start with an easier protocol to facilitate participation from the general public.

 - We included both Figure 2 and Figure 3 to provide two different representations of the same results. The differences in the tree are due to the different display of the dendrogram. The same three is underling based on the same distance (euclidian distance on log10 counts matrix, with ward clustering method).

 - The last comment is an interesting work hypothesis. We however do not know when these ingredients are added to the brewing process, and whether they were pre-treated before addition. If these ingredients contained living microorganisms at the moment of addition, they may indeed affect the brewing process and the taste of the final product. We believe collecting this metadata is outside the scope of this dataset description.

# Peer review: Referee response for version 1

**DOI:** 10.5256/f1000research.13607.r25906

**Published:** 2017-09-18

**Authors:** Matthew L. Bochman

**Affiliations:** 1 Molecular and Cellular Biochemistry Department, Indiana University Bloomington, Bloomington, IN, USA

In this manuscript, Sobel *et al*. present fungal microbiome data from 39 different beers as the culmination of a crowdfunded citizen science campaign.  These data will be of interest to citizen scientists and financial backers of the project, as well as those in the fermentation (especially beer) industry.  Overall, the data seem sound, but I have some concerns:

**Major comments**

 Were any controls for contamination used, *i.e.*, are all of the fungi identified actually from the beer samples?  The sequencing of a non-beer sample such as water that had been handled in the same way as the beer samples would help to determine if fungal DNA contamination occurred during sample processing.

 In line with the comment above, the manuscript states that “…microorganisms, or their DNA, could be carried over from the ingredients to the final product.”  Can the authors comment on whether they know if they are detecting the fungi themselves or DNA remnants from fungi that came from the raw ingredients of the beer?  Again, one could add purified control DNA to a mash, brew and bottle a beer, and then try to detect that DNA by PCR in the end product (or even at various stages along the brewing and fermentation process).  Attempting this with various concentrations of DNA would also yield information on how many cells of a particular species would be necessary on malted barley, for instance, to be detected in the final beer.

**Minor comments**

 In the introduction, the authors state that “…sour beer…[is] produced without the controlled addition of known yeast cultivates.”  Although this may be true for some types of sour beer like lambic and gueuze, many sour beers made in the U.S. are inoculated with known strains of yeast.  In those cases, the souring bacteria are usually the unknowns.

 Why was a fecal DNA prep kit used for DNA extraction?

 The authors collected 120 beers from 20 countries but only sequenced the fungi from 39 (mostly from Switzerland).  Is there an explanation for this attrition?

I have read this submission. I believe that I have an appropriate level of expertise to confirm that it is of an acceptable scientific standard, however I have significant reservations, as outlined above.

# Response

**Published:** 2017-10-10

**Authors:** Luc Henry

**Affiliations:** Hackuarium, Switzerland

Regarding major comments:

 - We did not include a water sample to account for any contaminant during the DNA extraction process. Processing such a control at this point would not reflect the original experimental conditions. We will include this control in the future when we will process new beers.

 - We did think of experiments to identify DNA remnants from raw ingredients but did not have the means to perform them. Indeed, the beer samples were sent from all over the world (Europe and Switzerland for the 39 reported in this data set) and we had no possibility to collect other related samples (raw ingredients, brewing environment, etc). We think that it is out of the scope of the current study. In this setup, it is not possible to know afterwards from which ingredient the DNA comes from and we comment on this in the text.

 Regarding minor comments:

 - We rephrased the sentence about sour beers accordingly.

 - Similar to soil and fecal material, beer samples were found to contain PCR inhibitors (see Juvonen and Haikara, J. Inst. Brew. 115(3), 167–176, 2009) that can interfere with the preparation of samples for sequencing analyses. The ZR Fecal DNA MiniPrep kit can overcome this because it provides filter columns designed to remove PCR inhibitors. This methodological choice was previously described by Bukolich and coworkers (eLife 2015;4:e04634 DOI: 10.7554/eLife.04634).

 - A majority of the samples we received were from industrial/filtered beers. Unfortunately the volumes we had at hand (typically a 330 mL bottle or below) did not yield enough material (DNA of good purity) to obtain sequencing results, as judged by QC of PCR products. In order to detect DNA in these beers, we would probably need to process a much larger volume of beer. We therefore did not include these samples in the final data set.

# Peer review: Referee response for version 1

**DOI:** 10.5256/f1000research.13607.r25904

**Published:** 2017-09-13

**Authors:** Bastian Greshake

**Affiliations:** 1 Department for Applied Bioinformatics, Institute for Cell Biology and Neuroscience, Goethe University, Frankfurt am Main, Germany

This article describes how innovative, participant-driven research projects can create an interesting data set outside the traditional Academy. This is an extremely laudable goal and the resulting data will be of interest to a broad audience. In its current state the article is a hybrid between data note, methods article and research article that delivers preliminary results. Regardless of the form of the article, the methods section would benefit a lot from a more detailed description of the methodology (see details below). Similarly, the analyses and results are a bit lacking at this stage, especially with respect to the basic metrics of the data sets (again, see below). 

**Major comments**

*Methods *

- the methods section should be significantly improved/extended for a better understanding. I'm aware that most/all the things are on *GitHub*, but having to crosslink these makes it hard to follow. Specifically the following things should be improved upon: 
  - What modifications were done to the protocol of the *ZR Fecal DNA MiniPrep *kit? (suggestion: putting the modified protocol to https://www.protocols.io/ if deemed useful)
  - Which parameters were used to perform the *bwa* alignment? 
  - What was the size of the reference database that was used for the mapping?
  - How was the hierarchical clustering done that is described in the methods section? Which clustering method was used? Which distance measure was used?

*Results*

- *"We obtained an average library size of 600K reads (min 350K, max 2400K)"* This is a rather large difference between the different libraries. Does the number of species found correlate with the sequencing depth? I.e. would you have found more species if you had sequenced more data for the smaller libraries? A rarefaction analysis would be useful to understand the impact of sequencing depth on species recovery. A minor, related suggestion: Having a table of sequencing statistics so that the reader can compare the samples.
- A major thing that is not mentioned in the results/discussion is the number of reads which could not be mapped against the reference database. How many reads of each library did not belong to any of the reference ITS sequences? And are the non-mapping reads similar to each other or can be clustered into OTUs? This would be needed to understand how many species/OTUs are in a given sample but could not be classified due to a lack of reference database. Without this the ITS diversity in a sample cannot be correctly estimated. 

**Minor comments:**

- *"we built the proof of concept for a targeted metagenome analysis pipeline for beer samples that can be used in high schools, citizen science laboratories, craft breweries or industrial plants" *It would be good to at least briefly discuss how this is currently limited by the need to have access to a high-throughput sequencer.
- It would be great if " *terroir*" could be defined in the introduction for those not too familiar with oenology
- *"a total of 88 fungal species were identified, including 52 unique occurrences"* are unique occurrences those species which are only found in a single beer? I'd suggest rephrasing it for a better understanding.
- *"Interestingly, most brews were found to contain low to medium presence of multiple other yeast species, including Saccharomyces bayanus (used in winemaking and cider fermentation), Saccharomyces kudriavzevii and Saccharomyces pastorianus (used in lager manufacturing), Saccharomyces eubayanus (a probable parent of Saccharomyces pastorianus) and Brettanomyces bruxellensis (typically used for the production of the Belgian beer styles)"* please include citations for these explanations of the different taxa.
- It's a matter of taste, but I recommend rethinking the use of *"microbial dark matter"*, c.f. http://merenlab.org/2017/06/22/microbial-dark-matter/ for an explanation of why.

I have read this submission. I believe that I have an appropriate level of expertise to confirm that it is of an acceptable scientific standard, however I have significant reservations, as outlined above.

# Response

**Published:** 2017-10-10

**Authors:** Luc Henry

**Affiliations:** Hackuarium, Switzerland

We thank the referee for his constructive remarks, and in particular for his comments on our methods section. We have added the details he requested (see below).

 Regarding major comments:

 - The minor modifications to the ZR Fecal DNA MiniPrep kit instructions are already described in the methods section of the article, as well as on the methods description available in the GitHub repository. These consist of, instead of starting with 50-100 mg of fecal material as suggested by the manufacturer, using a sludge pellet obtained through centrifuging 50 mL of beer (as described in the methods). Similar to soil and fecal material, beer samples were found to contain PCR inhibitors (see Juvonen and Haikara, J. Inst. Brew. 115(3), 167–176, 2009). The ZR Fecal DNA MiniPrep kit can overcome this because it provides filter columns designed to remove PCR inhibitors. This choice was previously described by Bukolich and coworkers (eLife 2015;4:e04634 DOI: 10.7554/eLife.04634).

 - In order to do the alignment we have used BWA (version 0.7.13 ) with standards parameters with 300 bp paired-end reads.

 - The ITS reference database contains 5361 ITS sequences. The average size of ITS is 585 bp with a standard deviation of 90 bp.

 - Hierarchical clustering was done by applying the basic Ward clustering algorithm with the euclidian distance computed on the log10 read count. We modified the legend of the Figure 2 accordingly.

 - We have to acknowledge the relatively large size variability between the different libraries. The amount of ITS detected can be affected by the sequencing depth and by the richness of the beer ecosystem. Accordingly, if the sequencing is not deep enough, we will clearly miss some low abundance species. If the beer sample contains a low variety of fungal species, sequencing deeper will not however provide additional information. In our analysis, it does not seem to be any correlation between the library size and the variety of ITS detected. The sample producing the largest library (2,4 mio reads, “Les Trois Dames”) is not the one with the largest detected ITS variety (11 species). Also, the beer sample containing the largest ITS diversity (“Waldbier 2014 Schwarzkiefer”, with 38 fungal species) had a library size of only about 0.35 mio reads. A rarefaction analysis is beyond the scope of this dataset description. As suggested, we added a table (Table 1) with the different informations regarding the libraries such as the mapping percentage and the total number of reads. In this table we observe that more than 99% of reads map to the ITS database. Consequently, there is a limited interest to try to find missing species or Operational Taxonomic Units (OTUs), and we can reasonably conclude that the ITS database used is comprehensive enough.

 Regarding minor comments:

 - The current data-set is a proof of concept that sequencing beer metagenomic information can be done, at least partly, with the help of the public. For the current analysis, we indeed had to rely on high-throughput sequencing technology available to us through a partnership with the genomic facility at the University of Lausanne. In the future, we would like to overcome this limitation, e.g. by using a minION sequencer. A remark was added to the discussion.

 - The text was modified to clarify the notion of "terroir".

 - The text was modified to clarify the notion of "unique occurrences".

 - The text was modified (species were removed) to reflect changes due to an updated sensitivity of the analysis (based on another referee's comment), and a reference was added.

 - The so-called “microbial dark matter” concept is regularly used by microbiologist and we think that it points to an interesting hypothesis worth mentioning, although it has nothing to do with the dark matter in physics, as explained in the blog post mentioned.
